# Analytical and clinical validation of the Lumipulse G plasma p‐tau217 assay for clinical implementation

**DOI:** 10.1002/alz.71374

**Published:** 2026-04-14

**Authors:** Burak Arslan, Johan Gobom, Ulf Andreasson, Laia Montoliu‐Gaya, Andrea L. Benedet, Anna Dittrich, Silke Kern, Ingmar Skoog, Nicholas J. Ashton, Tevy Chan, Nesrine Rahmouni, Pedro Rosa‐Neto, Kaj Blennow, Henrik Zetterberg, Hlin Kvartsberg

**Affiliations:** ^1^ Department of Psychiatry and Neurochemistry Institute of Neuroscience & Physiology, the Sahlgrenska Academy at the University of Gothenburg Mölndal Sweden; ^2^ Clinical Neurochemistry Laboratory, Sahlgrenska University Hospital Mölndal Sweden; ^3^ Department of Neuropsychiatry Region Västra Götaland, Sahlgrenska University Hospital Mölndal Sweden; ^4^ Banner Sun Health Research Institute Sun City Arizona USA; ^5^ Banner Alzheimer's Institute Phoenix Arizona USA; ^6^ Translational Neuroimaging Laboratory, Montreal Neurological Institute‐Hospital Montreal Canada; ^7^ The Research Institute of the McGill University Health Centre Montreal Quebec Canada; ^8^ Division of Geriatric Medicine Department of Medicine McGill University Montreal Quebec Canada; ^9^ Douglas Hospital Research Centre ‐ Centre intégré universitaire de santé et services sociaux de l'Ouest‐de‐l’Île‐de‐Montréal Verdun Quebec Canada; ^10^ Peter O'Donnell Jr. Brain Institute (OBI) University of Texas Southwestern Medical Centre (UTSW) Dallas USA; ^11^ Paris Brain Institute, ICM, Pitié‐Salpêtrière Hospital, Sorbonne University Paris France; ^12^ Neurodegenerative Disorder Research Center Division of Life Sciences and Medicine Department of Neurology Institute on Aging and Brain Disorders, University of Science and Technology of China and First Affiliated Hospital of USTC Hefei P.R. China; ^13^ Department of Pathology and Laboratory Medicine University of Wisconsin School of Medicine and Public Health Madison Wisconsin USA; ^14^ Wisconsin Alzheimer's Institute School of Medicine and Public Health, University of Wisconsin Madison Wisconsin USA; ^15^ Department of Neurodegenerative Disease Institute of Neurology University College London London UK; ^16^ UK Dementia Research Institute University College London London UK; ^17^ Hong Kong Center for Neurodegenerative Diseases Hong Kong China; ^18^ Centre for Brain Research, Indian Institute of Science Bangalore India

**Keywords:** Alzheimer's disease, analytical validation, lumipulse, plasma, p‐tau217

## Abstract

**INTRODUCTION:**

Although several plasma tau phosphorylated at threonine 217 (p‐tau217) immunoassays are now available, comprehensive analytical validation remains limited. We therefore evaluated the Lumipulse G plasma p‐tau217 assay and verified established cutoffs for clinical implementation.

**METHODS:**

This study was conducted in two phases: (1) analytical validation of the Lumipulse G plasma p‐tau217 assay, assessing precision, lower limit of quantification (LLoQ), selectivity, stability, and interference; and (2) verification of previously established cutoffs using a two‐threshold approach in 37 samples with confirmed cerebrospinal fluid (CSF) Aβ42/Aβ40 status.

**RESULTS:**

The assay demonstrated strong analytical performance, with repeatability and intermediate precision (%CV < 7%) and an LLoQ of 0.12 pg/mL. The p‐tau217 was stable across freeze–thaw cycles but less so at 4°C, and hemolysis > 2% introduced variability. Cutoff verification showed 97% reproducibility with excellent agreement (*ρ* = 0.99, *p* < 0.0001).

**DISCUSSION:**

The Lumipulse assay showed robust analytical performance and reproducibility, supporting clinical use, though certified reference materials are still needed for standardization.

## Highlights

- Transferring plasma samples to Hitachi cups improved analytical performance.
- Plasma samples should ideally be stored at −20°C or −80°C, rather than at 4°C.
- The Lumipulse plasma p‐tau217 assay demonstrated robust analytical performance, meeting all validation criteria, although samples with gross hemolysis or lipemia should be carefully handled.
- Plasma Lumipulse p‐tau217 showed excellent agreement with the high‐performing immunoprecipitation–mass spectrometry (IP‐MS) method.
- Predefined cutoffs were independently validated, supporting readiness for clinical use.

## 1 BACKGROUND

A definitive diagnosis of Alzheimer's disease (AD) still requires post‐mortem confirmation of extracellular amyloid‐β (Aβ) plaques and intracellular neurofibrillary tangles composed of hyperphosphorylated tau.^1^ In clinical practice, in vivo detection relies on amyloid and tau positron emission tomography (PET),^2^, ^3^ and cerebrospinal fluid (CSF) biomarkers,^4^, ^5^ several of which have received approval from the United States Food and Drug Administration (FDA) and^6^, ^7^ and European Medicines Agency (EMA) for their use in supporting the clinical diagnosis of AD.^8^, ^9^ However, their implementation is limited by the invasiveness of lumbar puncture and the cost, availability, and radiation exposure associated with PET imaging. These limitations have accelerated the development of blood‐based biomarkers as scalable alternatives. Blood sampling is minimally invasive, cost‐effective, and readily integrated into routine care.^10^ Advances in ultrasensitive technologies now allow reliable quantification of low‐abundance brain‐derived proteins in plasma, with several assays available on fully automated platforms suitable for high‐throughput clinical laboratory use.^11^, ^12^, ^13^, ^14^, ^15^

Among blood‐based biomarkers, phosphorylated tau (p‐tau) species are particularly promising for detecting AD‐related amyloid pathology, demonstrating greater disease specificity than Aβ peptides, whose circulating levels are influenced by peripheral production.^16^, ^17^ Of the different isoforms, plasma p‐tau217 consistently shows the highest diagnostic accuracy for early AD pathology,^18^, ^19^ with performance comparable to CSF.^12^, ^20^ In a recent Round Robin study, p‐tau217 showed larger fold‐changes and greater cross‐platform agreement than other p‐tau species.^16^ The Lumipulse G p‐tau217 assay exhibited the highest fold‐change among immunoassays, second only to immunoprecipitation–mass spectrometry (IP‐MS),^16^ contributing to the FDA approval of the plasma p‐tau217/Aβ42 ratio on this fully automated chemiluminescent platform.^21^ However, substantial differences in absolute concentrations across assays highlight the need for certified reference materials (CRMs) to enable harmonization.^16^

As laboratories move toward implementing plasma p‐tau217 in biomarker‐supported diagnostic algorithms with predefined cutoffs, careful evaluation of analytical performance becomes essential. Plasma p‐tau217 can be quantified using mass spectrometry (MS)^22^, ^23^ or immunoassay‐based platforms.^24^ Although MS offers high analytical specificity and accuracy,^24^ its routine use is limited by cost, complexity, and throughput constraints. Fully automated immunoassays, in contrast, provide scalability and operational practicality suited to clinical laboratories. Nevertheless, the absence of CRMs and assay‐specific differences in antibodies and calibrators contribute to variability in absolute concentrations across platforms,^16^ limiting interchangeability. Despite several head‐to‐head comparisons^24^, ^25^, ^26^ comprehensive analytical validation studies of widely used plasma p‐tau217 immunoassays remain limited.

In this context, we recently performed a formal analytical comparison of three widely used plasma p‐tau217 immunoassays (Lumipulse G, Meso Scale Discovery (MSD S‐Plex), and an in‐house single molecule array (Simoa) assay) available in our clinical laboratory using consecutively collected leftover clinical samples reflecting real‐world laboratory heterogeneity.^27^ This comparison was undertaken prior to selecting a platform for routine use, with the aim of directly assessing run‐to‐run variability, sample‐level analytical variation, and overall robustness across the assays available to us under routine laboratory conditions. This work demonstrated assay‐dependent systematic and proportional biases, differences in analytical variability, and limited interchangeability of absolute concentrations, underscoring the importance of assay‐specific validation prior to routine clinical implementation. Within this comparative framework, the Lumipulse platform showed high between‐run agreement and low sample‐level imprecision, supporting its selection as a candidate for routine implementation in our laboratory.

In this study, we selected the Lumipulse G plasma p‐tau217 assay for thorough analytical validation with the aim of implementation in routine clinical laboratory workflows, informed by multiple clinical diagnostic studies, including the plasma p‐tau Round Robin study^16^ and prior analytical method comparison study we performed in our clinical laboratory^27^ with the aim of implementation in routine clinical laboratory workflows. We further compared the assay with IP‐MS^28^ to assess its agreement with this high‐performing method. Finally, we independently verified previously established clinical cutoffs^14^ using a two‐cutpoint approach in a subset of well‐characterized cohort samples.

**RESEARCH IN CONTEXT**

1. **Systematic review**: The authors reviewed the literature using PubMed, focusing on publications from 2020 onward, which was determined as the point when phospho‐tau (p‐tau) species transitioned from cerebrospinal fluid to blood. Although several studies conducted full analytical validation of p‐tau217, no publication was found that performed comprehensive analytical validation of Lumipulse plasma p‐tau217.
2. **Interpretation**: Our results showed that Lumipulse plasma p‐tau217 demonstrated strong analytical performance, but samples with gross hemolysis should be carefully evaluated in routine clinical laboratory workflows. Its excellent agreement with the high‐performing immunoprecipitation–mass spectrometry (IP‐MS) method provides important additional evidence supporting its robust clinical performance.
3. **Future directions**: Despite the availability of multiple high‐performing plasma p‐tau217 assays for potential clinical implementation, differences in their absolute concentrations highlight the need for harmonization and standardization efforts. This will require the development of a reference method, followed by the creation of certified reference materials.

## 2 METHODS

### 2.1 Study design and participants

This study was conducted in two sequential phases. In the first phase, the Lumipulse plasma p‐tau217 assay underwent thorough analytical validation with the aim of routine implementation, with key performance characteristics evaluated, including repeatability, intermediate precision, lower limit of quantification (LLoQ), interferences, selectivity, and analyte stability. For this phase, anonymized leftover K_2_EDTA (dipotassium‐ethylenediaminetetraacetic acid) plasma samples—originally collected for routine analyses at the Neurochemistry and Clinical Chemistry Laboratories at Sahlgrenska University Hospital—were used. All samples were de‐identified, and no clinical or demographic information was available to the investigators. At the end of the first phase, we also assessed agreement between the Lumipulse plasma p‐tau217 assay and the high‐performing IP‐MS method using a subset of samples from the TRIAD cohort,^29^ in which amyloid positivity was determined by PET imaging. In the second phase, previously established cutoffs^14^ were independently validated in a smaller, well‐characterized sample subset from the Gothenburg H70 Birth Cohort Study,^30^ where amyloid positivity was defined by the CSF Aβ42/Aβ40 ratio. Details of these cohorts, including the definition of amyloid positivity and cutoffs used, have been described elsewhere.^29^, ^30^

### 2.2 Ethics approval and consent to participate

The collection and use of anonymized plasma samples at the Clinical Chemistry Laboratory, Sahlgrenska University Hospital, were approved by the Ethics Committee at the University of Gothenburg (EPN140811). The Gothenburg H70 Birth Cohort Study was conducted in accordance with the Declaration of Helsinki and approved by the Regional Ethical Review Board in Gothenburg (approval number: Dnr 869‐13). Written informed consent was obtained from all participants and/or their legal representatives prior to inclusion in the study. All TRIAD participants, or their legal representatives, gave written informed consent, and ethical approval for the study was granted by the Montreal Neurological Institute PET Working Committee and the Douglas Mental Health University Institute Research Ethics Board (MP‐18‐2017‐157).

### 2.3 Sample collection and preparation

For the analytical validation phase, only anonymized, randomly collected leftover K_2_EDTA plasma samples were used. For the assessment of the agreement between Lumipulse and IP‐MS p‐tau217, K_2_EDTA plasma samples from the TRIAD cohort were analyzed, while validation of the predefined cutoffs was performed using plasma samples from the Gothenburg H70 Birth Cohort Study. Prior to analysis, all study and quality control (QC) samples were thawed at room temperature, vortexed, and centrifuged, following the instructions provided in the respective assay manuals and the established protocol for the in‐house developed assay. Samples were centrifuged at 2000 × *g* for 10 minutes.

### 2.4 Lumipulse G plasma p‐tau217 protocol

This commercial assay was developed to quantitatively measure p‐tau217 in human plasma. The measurements were performed on a LUMIPULSE^®^ G 1200 platform according to kit insert instructions with the following modifications:

Blood samples were collected in 5 mL K_2_EDTA tubes and centrifuged within 3 h (10 minutes, 2000 × *g*). Plasma was transferred to polypropylene (PP) tubes (Sarstedt, 0.5 mL, REF 72.730.003) and stored at −80°C. On the day of analysis, samples were thawed at room temperature for 30–60 minutes, depending on sample volume. After thawing, samples were vortexed and centrifuged again (10 min, 2000 × *g*, room temperature). Supernatant was carefully pipetted from the middle of the tube to avoid lipemic layers and residual material and then transferred to Hitachi sample cup for final analysis.

An overview of the assay characteristics is presented in Supplementary Table 1.

### 2.5 Assay validation

The Lumipulse p‐tau217 assay was selected for validation based on supporting evidence from a recent Round‐Robin study,^16^ its strong performance,^14^ and prior analytical method comparison study we performed in our clinical laboratory^27^ as a candidate for routine implementation. The choice was further motivated by practical considerations, including workload efficiency, the user‐friendly design of the fully automated platform, and the growing evidence supporting its analytical accuracy and clinical utility^13^, ^14^, ^26^—a series of selected assay validation experiments were conducted in accordance with the recommendations outlined by Andreasson et al.^31^

#### 2.5.1 Comparison of sample cups

To evaluate whether the type of sample cups influenced assay analytical performance, plasma collected in standard tubes (Sarstedt, 0.5 mL, REF 72.730.003) was compared with instrument‐specific sample cups (Hitachi). A total of 100 samples were run sequentially in duplicate, and CV% was calculated.

#### 2.5.2 Repeatability and intermediate precision

Plasma leftovers from de‐identified patient samples were pooled to generate two in‐house QC materials representing low and high p‐tau217 concentrations. The low QC pool had an approximate concentration of 0.16 pg/mL, while the high QC pool had a concentration of approximately 0.31 pg/mL. Both pools were aliquoted into cryovials and stored at −80°C until analysis. In addition, two levels of manufacturer‐provided commercial QC materials were analyzed in parallel during each run. The low commercial QC had a target concentration of 0.495 pg/mL, and the high commercial QC had a concentration of 3.89 pg/mL. Each in‐house QC material was analyzed in quintuplicate across five separate days to assess repeatability and intermediate precision. Measurements were performed by a single technician using two different Lumipulse G instruments and two different reagent kit lots (cartridge lots 5022 and 5023; substrate lot 5061). Thus, between‐day, between‐instrument, and lot‐to‐lot variability were incorporated within the intermediate precision design. Lot‐to‐lot consistency following clinical implementation will continue to be monitored longitudinally under real‐world laboratory conditions as part of routine quality assurance procedures.

#### 2.5.3 Lower limit of quantification determination

**FIGURE 1:**
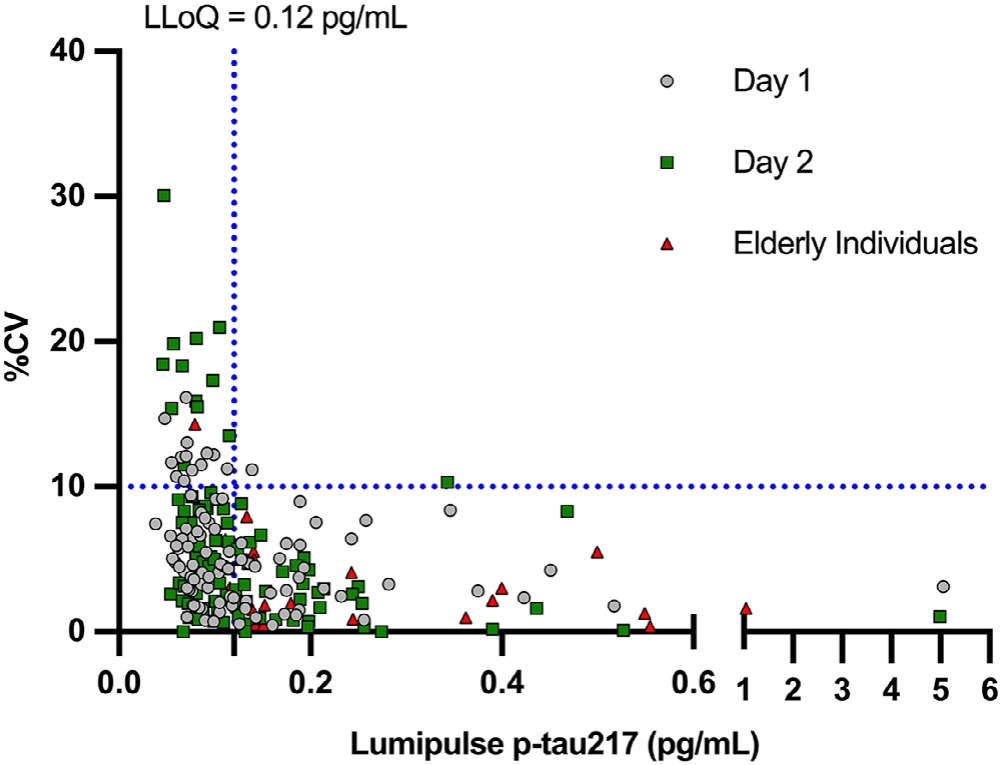
Determination of the functional LLoQ for the Lumipulse p‐tau217 assay. %CV values plotted against measured Lumipulse p‐tau217 concentrations (pg/mL) across two runs: Day 1 (gray circles), Day 2 (green squares), and samples from elderly individuals (red triangles). A horizontal dashed line at 10% CV and a vertical dashed line at 0.12 pg/mL are shown. The intersection of these thresholds defines the assay's LLoQ, estimated as 0.12 pg/mL. CV%, coefficient of variation; LLoQ, lower limit of quantification.

To establish the LLoQ for the Lumipulse p‐tau217 assay, the coefficient of variation (CV)% was calculated for each sample and plotted against the corresponding p‐tau217 concentration (Figure 1). A total of 122 plasma samples were included, comprising randomly collected 100 samples analyzed in duplicate across two separate days, and 22 additional samples from elderly individuals analyzed in duplicate on a single day. All were anonymized and randomly collected leftover samples received in our laboratory. The %CV was plotted on the y‐axis and p‐tau217 concentration (pg/mL) on the x‐axis. A horizontal threshold line was set at %10 CV to define acceptable analytical precision. A vertical line representing the LLoQ was drawn at the lowest concentration at which the %CV remained below this threshold in at least 95% of the cases.

#### 2.5.4 Interferences

Hemolysis was evaluated as a potential source of assay interference through two separate experiments. Briefly, two K_2_EDTA tubes were collected from each individual—one to obtain non‐hemolyzed plasma, and the other to induce hemolysis and generate hemolyzed plasma for spiking procedure. Detailed descriptions of these experiments are provided in the Supplementary Methods.

#### 2.5.5 Selectivity

To evaluate the selectivity of the assay, plasma samples from five individuals were spiked with in‐house produced non‐phosphorylated full‐length (i.e., 441 amino acid‐long) recombinant tau protein (tau‐441). As it is currently not feasible to directly assess the assay's selectivity for p‐tau217 over other phosphorylated tau isoforms, this experiment was designed to determine whether the p‐tau217 assay exhibits cross‐reactivity with non‐phosphorylated tau species. Briefly, recombinant non‐phosphorylated tau‐441 protein (concentration: 0.7  mg/mL) was initially diluted 1:100 in phosphate‐buffered saline (PBS), followed by a second 1:100 dilution to achieve a final concentration of 70 pg/µL. This solution was then further diluted 1:70 using Lumipulse sample diluent to obtain a working stock concentration of 1 pg/µL. Serial dilutions were subsequently prepared to generate a range of spiking concentrations (Table 1 and Supplementary Table 2). For the final spiking step, 5 µL of each dilution was added to 250 µL of plasma, and the resulting samples were analyzed using the Lumipulse p‐tau217 assay.

**TABLE 1:** Evaluation of the Lumipulse p‐tau217 assay selectivity against non‐phosphorylated Tau‐441 protein.

Tau‐441 Spike‐in (pg/mL)	Sample 1 (pg/mL)	Sample 2 (pg/mL)	Sample 3 (pg/mL)	Sample 4 (pg/mL)	Sample 5 (pg/mL)
20	0.323	1.118	0.87	0.771	0.329
10	0.348	1.135	0.9	1.356	0.305
5	0.329	1.068	0.881	0.76	0.329
2	0.324	1.091	0.914	1.564	0.324
1	0.355	0.976	0.986	1.58	0.335
0	0.323	0.922	0.846	0.929	0.322

*Note*: Plasma from five individuals was spiked with recombinant non‐phosphorylated Tau‐441 with different concentrations.

#### 2.5.6 Sample stability

Sample stability was tested under three different conditions: cold storage (4°C), frozen storage (−20°C), frozen storage (−80°C).

#### 2.5.7 Cold storage

To assess plasma stability during cold storage, leftover plasma samples from three individuals were aliquoted into two tubes per individual. One aliquot was immediately stored in an ultra‐freezer at −80°C to serve as the reference. The second aliquot was kept at cold storage conditions (2–8°C) for 3 days. After the cold storage period, these aliquots were transferred to an ultra‐freezer at −80°C until analysis.

#### 2.5.8 Frozen storage (−20°C)

Frozen storage stability at −20°C was evaluated using leftover plasma samples from five individuals. For each individual, plasma was aliquoted into four tubes. One aliquot was immediately stored at −80°C to serve as the reference. The remaining three aliquots were subjected to one, two, or three freeze–thaw cycles, respectively. For each cycle, samples were stored at −20°C for the designated number of cycles before being transferred to an ultra‐freezer at −80°C for storage until analysis.

#### 2.5.9 Frozen storage (−80°C)

Frozen storage stability at −80°C was evaluated using leftover plasma samples from five individuals. For each individual, plasma was aliquoted into four tubes. One aliquot was immediately stored at −80°C to serve as the reference. The remaining three aliquots were subjected to one, two, or three freeze–thaw cycles, respectively. For each cycle, samples were stored at −80°C for the designated number of cycles before being transferred to an ultra‐freezer at −80°C for storage until analysis.

#### 2.5.10 Cutoff validation

To validate pre‐defined clinical cutoffs using a two‐cutpoint approach for plasma p‐tau217 established and published elsewhere,^14^ we applied the previously defined thresholds (< 0.22 pg/mL for low probability, 0.22–0.34 pg/mL as intermediate probability, and >0.34 pg/mL for high probability of AD‐related brain amyloidosis) to an independent cohort of participants with known amyloid pathology status. The p‐tau217 concentrations were categorized according to these cutoffs, and any shift in risk classification between the original and validation runs was assessed.

#### 2.5.11 Agreement between IP‐MS and Lumipulse plasma p‐tau217

Although the recent Round Robin study^16^ supported and motivated the use of Lumipulse plasma p‐tau217 in routine practice, we also evaluated its agreement and performance against IP‐MS in a subset of the TRIAD cohort (*n* = 54) comprising samples across the AD continuum. The IP‐MS p‐tau217 method has been described in detail elsewhere.^28^

### 2.6 Statistical analysis

All statistical analyses were performed using MedCalc Statistical Software version 23.1.3 (MedCalc Software, Ostend, Belgium) and GraphPad Prism version 9.5.1 (GraphPad Software, San Diego, CA, USA). Prior to analysis, data distribution and normality were assessed using the D'Agostino–Pearson test. As the data were not normally distributed, non‐parametric tests were applied in subsequent analyses. To compare measurements from paired samples analyzed on different days, the Wilcoxon matched‐pairs signed‐rank test was used. The correlation between runs was assessed using Spearman's rank correlation coefficient. Intra‐assay precision was expressed as the CV%, calculated as: CV% = (standard deviation/mean) × 100 for replicate measurements of each sample. Mann–Whitney *U* test was used to compare measured p‐tau217 concentrations between different instruments and between different reagent LOTs at low and high concentration levels. For the comparison and correlation of the reference and validation runs the Wilcoxon matched‐pairs signed‐rank test and Spearman's rank correlation were used. To compare Lumipulse and IP‐MS, fold‐change values for p‐tau217 were calculated using the average biomarker levels in the cognitively unimpaired (CU−) group as the reference across the AD continuum. Diagnostic accuracy for differentiating amyloid pathology status was assessed for both Lumipulse and IP‐MS using the area under the receiver operating characteristic curve (AUROC). In addition, the Youden index was applied to estimate cutoffs for distinguishing amyloid‐positive and amyloid‐negative groups in the whole sample set. *p*‐Value < 0.05 was considered statistically significant for all analyses.

## 3 RESULTS

### 3.1 Comparison of sample cups

In our initial experiment, we ran 100 plasma samples in their original collection tubes (Sarstedt) after centrifugation and observed a relatively high mean CV% of 10.68% (mean p‐tau217: 0.26 pg/mL). Following this, we transferred the plasma manually into Hitachi sample cups after centrifugation, which resulted in improved precision, with a reduced mean CV% of 6.42% (mean p‐tau217: 0.20 pg/mL). Based on these findings, we proceeded to run all subsequent samples in Hitachi cups.

### 3.2 Analytical reproducibility for clinical samples – Lumipulse G

To assess analytical variation in clinical samples, plasma aliquots from 100 individuals—selected to cover the full range of p‐tau217 concentrations—were analyzed on two separate days using the Lumipulse G assay (with 1 week between replicates). Although a statistically significant difference was observed between runs (*p* < 0.001), a strong correlation was found (*ρ* = 0.98). Intra‐assay precision was further evaluated, with the mean CV% of duplicate measurements calculated as 6.42%. Detailed results are provided in the Supplementary File (Supplementary Figures 1 and 2).

### 3.3 Repeatability and intermediate precision of Lumipulse G

Repeatability and intermediate precision were determined by analyzing aliquots of a low (0.16 pg/mL) and a high (0.31 pg/mL) QC sample for p‐tau217, prepared by pooling plasma from several individuals. A summary of the results is shown in Table 2. For the low QC sample, the CV for the repeatability (within‐day variation) was 5.43% and the CV for the intermediate precision (between‐day) was 6.96%. For the high QC sample, the repeatability was 4.12% and the intermediate precision was 5.57%. All precision results remained well within the pre‐specified acceptance criterion of 10%. Additionally, commercial quality control materials analyzed alongside the study QCs consistently demonstrated CV values within 5%, further supporting the robustness and stability of assay performance during the precision experiments. The expanded measurement uncertainty, calculated with a coverage factor of 2, is based on the highest observed repeatability (CV_R_) (5.43%). This results in an expanded measurement uncertainty of approximately 11%, calculated using the formula:

2×5.43%≈11%

**TABLE 2:** Repeatability and intermediate precision of plasma Lumipulse p‐tau217.

			Repeatibility, r		Intermediate precision, Rw	
Parameter	Sample	Mean (pg/mL)	SD_r_	%CV_r_	SD_Rw_	%CV_Rw_
Pool	Low	0.156	0.008	5.43	0.011	6.96
	High	0.306	0.013	4.12	0.017	5.57
Commercial	Low	0.495	0.015	3.01	0.027	5.45
	High	3.89	0.080	2.05	0.124	3.2

*Note*: Two levels of pooled plasma samples were used for precision experiments, as detailed in the Methods section. “Commercial” refers to quality control materials provided by the manufacturer. “Mean” indicates the mean concentration of the sample pool for plasma at each of the three levels. “%CV_r_” represents repeatability, while “%CV_Rw_” represents intermediate precision.

Abbreviation: CV, coefficient of variation; SD, standard deviation.

This value was further used to define an acceptable range of variability, such as in sample stability experiments.

### 3.4 LLoQ determination

The LLoQ was conservatively set at 0.12 pg/mL, the lowest concentration at which at least 95% of samples showed a CV ≤ 10%, as presented in Figure 1. At this level, the assay provided reliable quantification with acceptable precision under routine laboratory conditions.

### 3.5 Interferences – hemolysis

**FIGURE 2:**
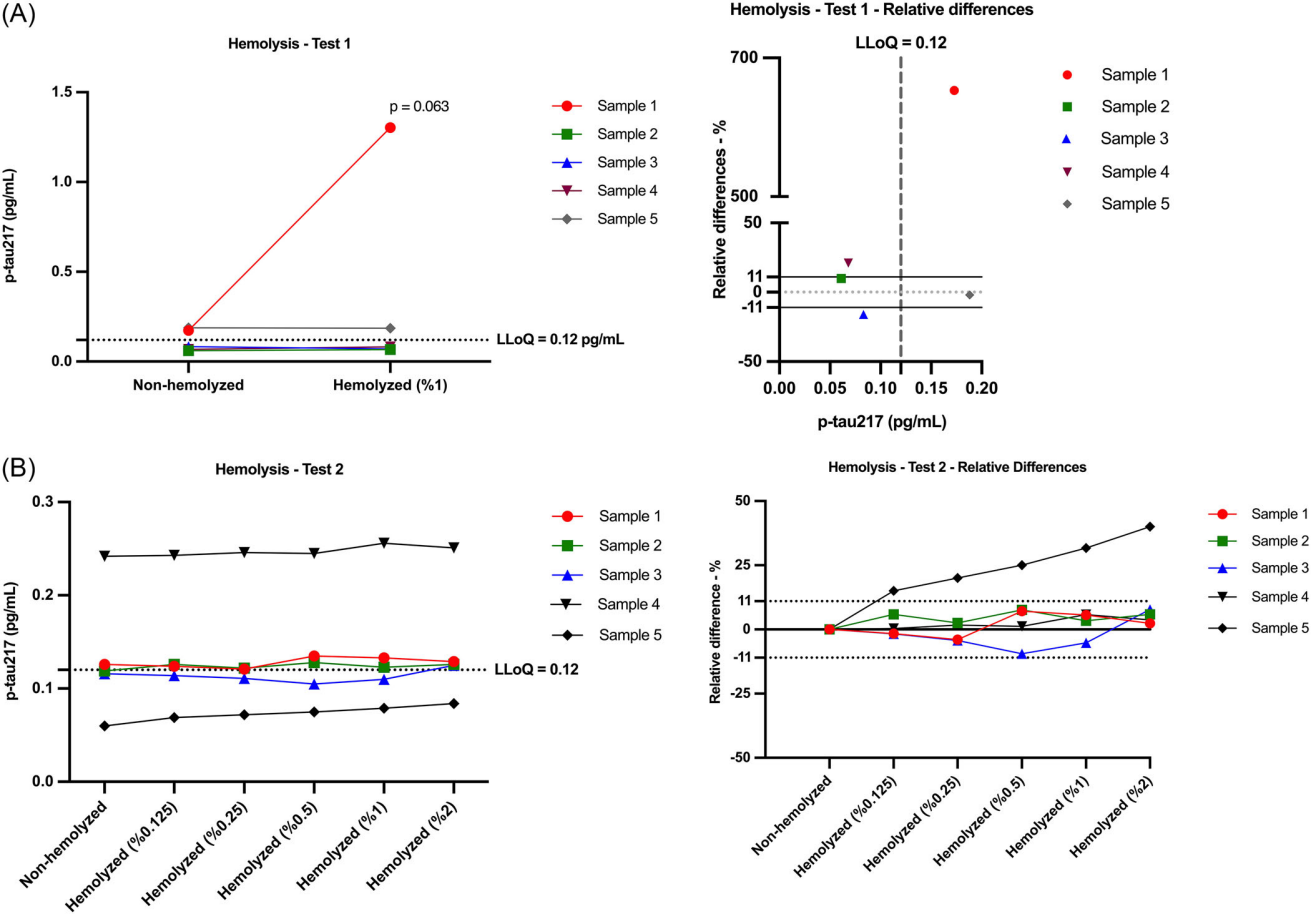
Evaluation of the effect of hemolysis on p‐tau217 concentrations across two independent experiments. (A) In the first experiment, plasma samples from five individuals were spiked with 1% hemolyzed plasma to evaluate assay sensitivity to hemolysis. The left panel shows individual p‐tau217 concentrations before and after spiking, with one sample (Sample 1) showing a substantial increase. The right panel displays relative differences (%) compared to the non‐hemolyzed condition, highlighting variability across samples. Notably, Sample 1 (red) exhibited a > 600% increase and had a high lipemia index (66), while others showed negligible to moderate differences (≤ ± 25%). (B) In the second experiment, five visually non‐turbid plasma samples with low baseline hemolysis and lipemia indices were spiked with hemolyzed plasma at increasing concentrations (0.125%–2%). The left panel shows absolute p‐tau217 concentrations, and the right panel presents relative differences (%) from the non‐hemolyzed condition. Four of five samples remained stable across conditions (≤ ± 10% variation), while one sample (Sample 5) demonstrated a dose‐dependent increase. However, this sample's baseline concentration was below half the assay's LLoQ (0.12 pg/mL), and even at 2% spiking, measured values remained below the quantifiable range. LLoQ, lower limit of quantification.

In the first hemolysis experiment, plasma samples from five individuals were spiked with 1% hemolyzed plasma to evaluate the effect of hemolysis on p‐tau217 measurements. Two of the five samples showed negligible impact (relative differences ≤ ± 10%), with both pre‐ and post‐spike concentrations below the in‐house determined LLoQ (0.12 pg/mL). Another two samples showed a moderate effect (± 11%–25%); in one of these, both concentrations were below the LLoQ. The remaining sample exhibited a pronounced change (> ± 50%), which was confirmed upon re‐analysis (Figure 2A). Notably, this sample had visible turbidity and a high lipemia index (66) prior to the addition of hemolyzed plasma, whereas the lipemia index for the other samples was below 30. This finding suggests that elevated lipemia may exacerbate the assay's susceptibility to hemolysis‐related interference. Based on these findings, a second hemolysis experiment was conducted to further investigate the potential interaction between hemolysis and lipemia in only non‐turbid samples.

In the second experiment, five out of fifteen plasma samples were selected based on having the lowest hemolysis and lipemia indices and being visually clear and non‐turbid. These selected samples were spiked with hemolyzed plasma from the same individual at concentrations of 0.125%, 0.25%, 0.5%, 1%, and 2%. In summary, four of the five samples showed negligible impact (≤ ± 10%) across all spiking concentrations. One sample demonstrated a moderate (± 11%–25%) to substantial (± 26%–50%) effect, with a dose‐dependent trend—greater interference observed at higher hemolysis levels. However, this particular sample had a baseline p‐tau217 concentration below half of the assay's LLoQ, and even after 2% hemolysis spiking, the measured concentration remained well below the LLoQ (Figure 2B). An overview of the hemolysis experiments, along with the HIL Index (hemolysis, icterus, lipemia), is presented in Supplementary Table 3.

### 3.6 Selectivity

**FIGURE 3:**
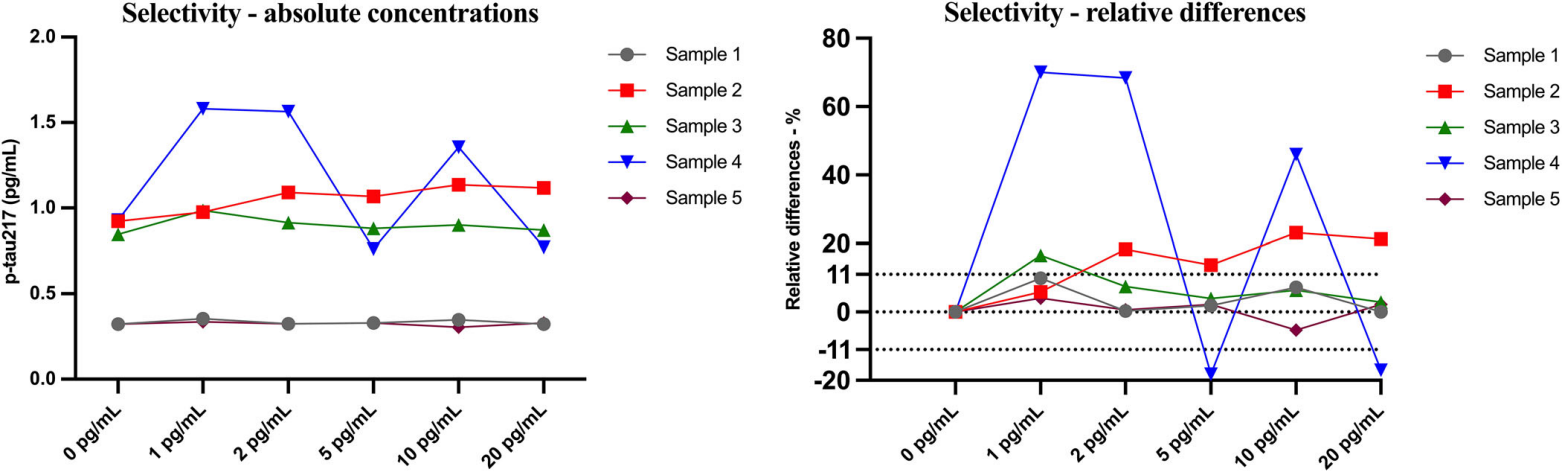
Selectivity assessment of the p‐tau217 assay using non‐phosphorylated recombinant Tau‐441. Selectivity evaluation of the p‐tau217 assay by spiking plasma samples from five individuals with increasing concentrations of non‐phosphorylated full‐length recombinant Tau‐441 protein (0.05 to 20 pg/mL). Absolute p‐tau217 concentrations remained largely stable across all spiking concentrations. Relative percentage differences from baseline (0 pg/mL) showed minimal variability and no dose‐dependent pattern, indicating no detectable cross‐reactivity.

Spiking with non‐phosphorylated full‐length recombinant Tau‐441 protein did not lead to consistent or dose‐dependent increases in measured p‐tau217 concentrations across the five plasma samples tested. Three out of five samples showed minor changes within the ±11% expanded uncertainty range. One sample exhibited markedly elevated p‐tau217 concentrations in some, but not all, spiked conditions, and another showed an 18.3% difference, though without a systematic dose–response trend. In summary, the changes in measured values were not proportional to the spike‐in concentrations. No consistent pattern indicative of cross‐reactivity was observed. The assay did not respond to non‐phosphorylated tau even at concentrations up to 20 pg/mL. Most samples remained close to baseline levels, supporting the conclusion that the method is not affected by non‐phosphorylated tau and demonstrates high specificity for tau phosphorylated at threonine 217 (Table 1, Figure 3).

### 3.7 Sample stability

**FIGURE 4:**
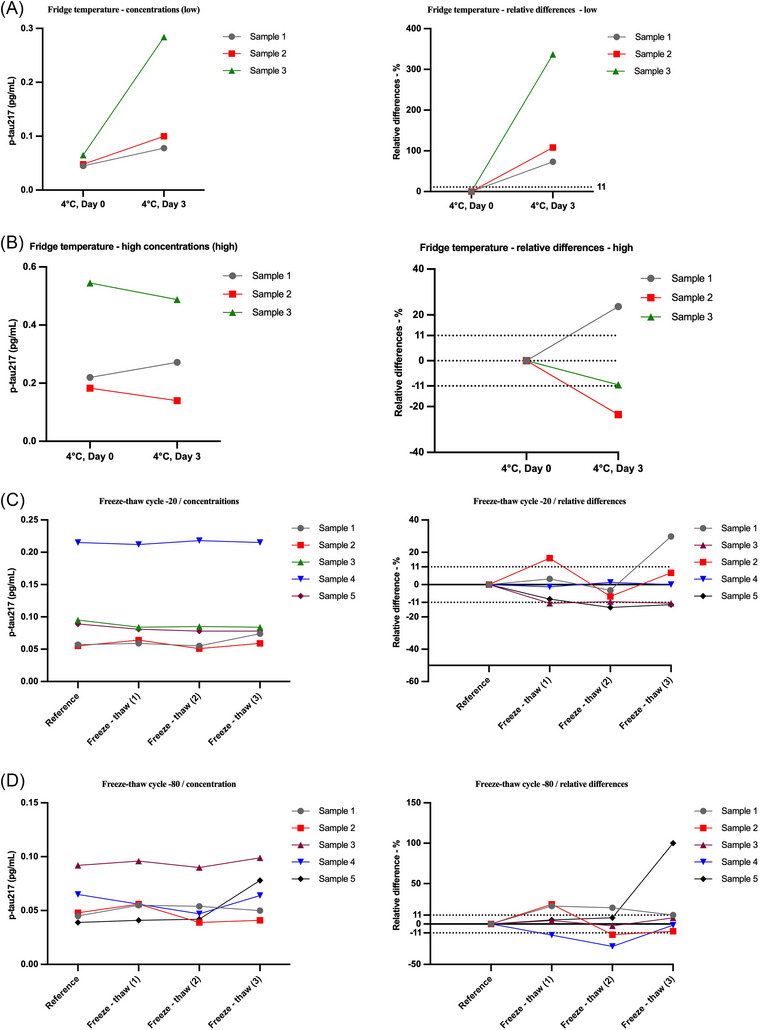
Evaluation of plasma p‐tau217 stability under different storage conditions. (A,B) Plasma samples stored at 4°C for up to 3 days showed concentration‐ and sample‐dependent variability. In low‐concentration samples (A), substantial increases were observed in some cases, exceeding the ±11% expanded uncertainty threshold. In contrast, samples with higher baseline concentrations (B) exhibited improved stability, although some variation still exceeded acceptable limits. (C,D) Plasma samples subjected to repeated freeze–thaw cycles at −20°C (C) and −80°C (D) demonstrated good stability. Most p‐tau217 concentrations remained within ± 11% of the reference value. Slightly greater variability was observed at −20°C, but the differences remained relatively minor and consistent. These results suggest that refrigerated storage is suboptimal for maintaining p‐tau217 integrity, particularly at low concentrations, while frozen storage at −80°C offers better stability for reanalysis and long‐term storage.

Plasma p‐tau217 concentrations demonstrated good stability under freeze–thaw conditions but showed substantial instability during refrigerated storage up to 3 days. For samples subjected to repeated freeze–thaw cycles at −80°C, most measurements remained within an expanded measurement uncertainty of ± 11%, indicating good stability (Figure 4D). Similarly, samples exposed to freeze–thaw cycles at −20°C showed relatively stable p‐tau217 concentrations, although slightly greater variability was observed compared to −80°C storage, with most differences still within ± 11% (Figure 4C). In contrast, initial experiments revealed marked instability during refrigerated storage at 4°C, with samples exceeding the ± 11% limit (Figure 4A). However, in a follow‐up experiment using samples with relatively high p‐tau217 concentrations, the overall stability during 3‐day storage at 4°C was notably improved. Although some samples still showed deviations beyond ± 11%, the magnitude of change was smaller and more consistent compared to earlier tests (Figure 4B). These findings suggest that refrigerated storage is not ideal for preserving sample integrity, particularly if samples are intended for reanalysis. Instead, freezing samples is recommended to ensure the stability of p‐tau217 over time.

### 3.8 Cutoff validation

Pre‐defined risk‐based cutoffs for plasma p‐tau217 (<0.22 pg/mL for low risk, 0.22–0.34 pg/mL for intermediate risk, and > 0.34 pg/mL for high risk of AD‐related brain amyloidosis)^14^ were applied to a cohort of 37 individuals with known amyloid pathology status to assess their diagnostic utility and technical robustness. Results from an initial measurement run *“reference”* and a second independent run *“validation”* were compared to evaluate reproducibility of classification. Out of the 37 samples, 36 (97%) retained the same risk category between runs. A single sample shifted from the intermediate‐risk group (0.23 pg/mL) in the original run to the low‐risk group (0.20 pg/mL) in the validation run, reflecting a minor change near the cutoff threshold of 0.22 pg/mL.

Risk group distributions and amyloid positivity for each run are presented in Table 3. In both runs, the high‐risk group included 18/18 amyloid‐positive individuals (100% true positive rate), while the low‐risk group contained only amyloid‐negative individuals (100% true negative rate). As expected, the intermediate‐risk group remained diagnostically mixed. Comparative analysis of numerical p‐tau217 concentrations showed no significant difference between runs (*p* = 0.40). The method demonstrated excellent correlation across runs (*ρ* = 0.99, *p* < 0.0001) (Supplementary Figure 3). Median p‐tau217 levels were comparable (0.34 pg/mL vs. 0.33 pg/mL) (Supplementary Figure 3), and the range, mean, and variability remained consistent, underscoring the assay's reproducibility under routine conditions.

**TABLE 3:** Plasma p‐tau217 classification according to risk‐based cutoffs in two independent analytical runs.

Parameter	Risk group	No. of individuals	Amyloid positivity (n)	p‐tau217 (pg/mL)
Reference run	High probability	18	18	0.66 (0.42 – 1.64)
	Intermediate probability	9	6	0.30 (0.23 – 0.34)
	Low probability	10	0	0.12 (0.08 – 0.18)
Validation run	High probability	18	18	0.69 (0.43 – 1.62)
	Intermediate probability	8	6	0.29 (0.23 – 0.33)
	Low probability	11	0	0.13 (0.09 – 0.20)

*Note*: Plasma p‐tau217 concentrations and CSF Aβ42/Aβ40 status of 37 individuals classified using pre‐defined risk‐based cutoffs in two independent assay runs (reference and validation). p‐tau217 concentrations are presented as median (range) for each group. Individuals were stratified into high (> 0.34 pg/mL), intermediate (0.22–0.34 pg/mL), and low (< 0.22 pg/mL) risk categories based on their p‐tau217 values. Classification remained consistent between runs for 36 out of 37 individuals (97%). A single sample initially classified as intermediate risk (0.229 pg/mL) in the reference run shifted to low risk (0.204 pg/mL) in the validation run, a change occurring near the decision threshold.

Abbreviations: p‐tau217, phosphorylated tau at threonine 217; CSF, cerebrospinal fluid.

### 3.9 Agreement between IP‐MS and Lumipulse plasma p‐tau217

Demographics and clinical characteristics of the TRIAD cohort are presented in Supplementary Table 4. In a subgroup analysis of samples from 54 participants (female *n* = 26, 48.1%), the correlation between Lumipulse and IP‐MS p‐tau217 was very strong (*ρ* = 0.92, *p* < 0.0001). Average fold‐changes across clinical groups were broadly similar, and both assays showed excellent accuracy for detecting amyloid PET positivity, with nearly identical AUCs (Lumipulse = 0.99, IP‐MS = 0.98). Full results are presented in Table 4.

**TABLE 4:** Performance metrics and agreement between Lumipulse G and IP‐MS plasma p‐tau217 assay.

	Average fold mean (SD)							Accuracy to detect amyloid PET positivity				
Assay	CU+ (*n* = 10)	MCI+ (*n* = 27)	ADD (*n* = 4)	MCI‐ (*n* = 3)	Spearman Correlation (95%CI)	AUC (95%CI)	Sensitivity (95%CI)	Specificity (95%CI)	Accuracy (95%CI)	PPV (95%CI)	NPV (95%CI)	Cutoff
Lumipulse	3.61 (2.13)	6.38 (3.68)	13.48 (8.38)	1.14 (0.44)	0.92 (0.86 to 0.95)	0.99 (0.94 to 1.0)	95.1 (83.5 to 99.4)	100 (75.3 to 100)	96.3 (87.3 to 99.5)	100	86.7 (62.7–96.2)	0.14
IP‐MS	3.67 (2.77)	4.90 (2.11)	9.33 (6.35)	1.70 (0.56)		0.98 (0.91 to 1.0)	82.9 (67.9 to 92.8)	100 (75.3 to 100)	87.0 (78.1 to 96.0)	100	65.0 (48.6–78.5)	0.004

*Note*: Fold means were calculated using the CU− group (*n* = 10) as the reference. Accordingly, the average value for this group was set to 1 and is not displayed in the table.

Abbreviations: ADD, Alzheimer's disease dementia; AUC, area under the curve; CI, confidence interval; CU, cognitively unimpaired; CV, coefficient of variation; MCI, mild cognitively impaired;NPV, negative predictive value; PPV, positive predictive value; SD, standard deviation.

## 4 DISCUSSION

Previous studies have primarily focused on the diagnostic accuracy of Lumipulse plasma p‐tau217 and/or its ratio with Aβ42, whereas comprehensive analytical validation has been limited. Data on agreement between plasma IP‐MS and Lumipulse p‐tau217 are also scarce, despite their importance for future CRM development and assay recalibration. In this two‐phase proof‐of‐concept study, we first performed a structured analytical validation of the Lumipulse plasma p‐tau217 assay. The Lumipulse platform was selected based on a combination of prior in‐house analytical method comparisons performed under real‐world laboratory conditions^27^ and supporting evidence from external comparison studies, including the plasma p‐tau Round Robin study,^16^ rather than relying on a single source of evidence. Considerations related to low within‐ and inter‐run variability, full automation, high throughput, and compatibility with existing laboratory workflows further supported its prioritization for potential clinical implementation. With few exceptions—most notably hemolysis interference in turbid samples and reduced stability at 4°C—performance metrics were within acceptable limits. We also independently verified previously established clinical cutoffs using a two‐cutpoint approach in a well‐characterized subset, with 36 of 37 samples (97%) retaining identical risk classification across runs. In addition, Lumipulse showed excellent agreement with IP‐MS, with nearly identical diagnostic performance.

To further support clinical implementation, we directly compared Lumipulse with IP‐MS^22^, ^28^, ^32^, ^33^ in samples analyzed by both methods. The assays demonstrated strong concordance, similar diagnostic accuracy, and comparable fold‐changes across the AD continuum, consistent with prior reports.^16^ Based on these supporting findings, we proceeded with the Lumipulse G plasma p‐tau217 assay for routine implementation.

As part of the pre‐analytical evaluation, we first examined whether sample cup type affected performance. Sarstedt polypropylene tubes initially showed occasional elevated CV%. When samples were transferred to Hitachi cups, precision improved, likely due to improved pipetting efficiency and reduced interference from residual material or lipid layers. Hitachi cups were therefore adopted for routine use.

In terms of precision, repeatability, and intermediate precision were robust, with %CVs below 5.43% and 6.96%, respectively. Although the manufacturer defines a measuring range of 0.03–10 pg/mL, we conservatively defined an LLoQ of 0.12 pg/mL based on a 10% CV threshold and intended clinical use.

Furthermore, the impact of hemolysis on plasma p‐tau217 concentrations was explored through two experiments, each revealing variable outcomes depending on the degree of hemolysis and inter‐individual variability. In the first experiment, four out of five samples showed negligible to moderate impact following the addition of 1% hemolyzed plasma. In contrast, one sample exhibited a substantial increase in p‐tau217 levels and was notable for its visible turbidity and markedly elevated lipemia index. To further investigate the potential interaction between hemolysis and lipemia, a second experiment was conducted using five plasma samples with the lowest hemolysis and lipemia indices, all of which were visually clear and non‐turbid. These samples were spiked with increasing concentrations of hemolyzed plasma (0.125%–2%). Four of the five samples remained unaffected across all conditions, while one sample demonstrated a dose‐dependent increase in p‐tau217. However, this particular sample had a baseline concentration below half of the assay's LLoQ, and even after 2% spiking, the measured value remained below the quantifiable range. While such effects have not been extensively reported for plasma p‐tau217, prior investigations on other phosphorylated tau species, such as p‐tau181, have yielded mixed results. One study reported a strong correlation between hemolysis and elevated p‐tau181 levels,^34^ suggesting a direct and predictable impact. Conversely, another study found no significant changes in p‐tau181 even at higher hemolysate concentrations.^35^ Collectively, these findings highlight that p‐tau immunoassays may be variably affected by hemolysis depending on the target epitope, analyte concentration, and matrix characteristics. In our study, the Lumipulse p‐tau217 assay appeared largely robust to low levels of hemolysis; however, individual sample factors—such as high lipemia or very low baseline p‐tau217—may increase susceptibility to interference. These observations support the importance of monitoring pre‐analytical variables like hemolysis and lipemia, especially in clinical and research settings where assay precision is critical.

Selectivity testing further demonstrated high assay specificity. Spiking plasma with non‐phosphorylated Tau‐441 (up to 20 ng/mL) produced no systematic increase in p‐tau217, indicating no cross‐reactivity and specificity for phosphorylation at threonine 217. To our knowledge, this is the first report examining selectivity of the Lumipulse plasma p‐tau217 assay.

In addition, our analyte stability experiments demonstrated that plasma p‐tau217 concentrations are well preserved under frozen conditions, supporting the suitability of both −80°C and −20°C storage for repeated freeze–thaw cycles. Most measurements remained within the acceptable ± 11% variation range, with only minor differences observed between the two temperatures. These findings are consistent with previous reports that assessed similar parameters using different assays for the same analyte.^36^, ^37^, ^38^ In contrast, refrigerated storage at 4°C resulted in notable instability in initial tests, with several samples showing deviations beyond acceptable limits. A subsequent experiment using higher p‐tau217 concentrations showed improved performance under 4°C storage, although some variability persisted. These findings suggest that, while short‐term refrigerated storage may be tolerable under certain conditions, frozen storage is strongly preferred to maintain assay integrity, particularly for samples intended for reanalysis or long‐term use. Establishing clear pre‐analytical guidelines for storage conditions will be essential to ensure the reliability of p‐tau217 measurements in clinical and research settings.

The application and validation of pre‐defined risk‐based cutoffs for plasma p‐tau217^14^ demonstrated excellent reproducibility and diagnostic consistency across two independent measurement runs. With 97% of samples retaining their risk classification, and perfect agreement in both the high‐risk (100% amyloid‐positive) and low‐risk (100% amyloid‐negative) groups, the cutoff strategy proved both robust and clinically informative. The intermediate‐risk range, as expected, showed more heterogeneity. High correlation and consistent descriptive statistics between runs further support the assay's technical reliability for stratifying individuals by AD‐related amyloid burden.

In contrast to p‐tau217, as previously noted, it is well established that Aβ in plasma is not only produced in the brain but also in platelets and peripheral tissues, as exemplified by the complete lack of correlation between plasma and CSF levels of either Aβ42 or Aβ40.^39^ As a result, the marked decrease in CSF Aβ42 levels associated with brain amyloid pathology is obscured in plasma by peripherally produced Aβ42 peptides. This confounding factor is method‐independent and results in the plasma Aβ42/Aβ40 ratio decreasing by only around 10% in amyloid PET‐positive cases, with marked overlap seen in healthy elderly individuals,^40^ in contrast to the clear bimodal distribution seen for the Aβ42/Aβ40 ratio in CSF.^41^ In clinical practice, this would also introduce a robustness issue, meaning that any type of variability (pre‐analytical, analytical, or batch‐to‐batch) would negatively affect this biomarker,^42^, ^43^ increasing the risk of misclassification. In addition, both plasma Aβ42 and Aβ40 are sensitive to medications that are commonly used in the elderly population, such as neprilysin inhibitors.^44^ Importantly, the IFCC Working Group on Biomarkers of Neurodegenerative Diseases (WG‐BND) has initiated standardization and harmonization efforts for plasma p‐tau217, including the development of a CRM. In contrast, similar efforts for plasma Aβ42 were not recommended due to the poor correlation observed between plasma methods. Given these confounding factors and limitations, and even though the p‐tau217/Aβ42 ratio has received FDA approval, the p‐tau217/Aβ42 ratio was not included in this study.

This study has some limitations. First, the analytical evaluation focused on the platforms available in our laboratory at the time of implementation and therefore did not include head‐to‐head comparisons with other fully automated assays, such as the Beckman Coulter or Roche Elecsys p‐tau217 assays. In addition, the analytical comparison was performed using unselected leftover clinical samples rather than well‐characterized research cohorts, as the primary aim was to assess analytical performance under real‐world clinical laboratory conditions. In addition, the sample size for evaluating agreement between the Lumipulse G immunoassay and the IP‐MS method was relatively limited, as IP‐MS requires large sample volumes, restricting the availability of plasma suitable for parallel measurements. Nevertheless, the observed fold changes were comparable to those reported in the recent plasma p‐tau Round‐Robin study,^16^ providing supportive external context for our findings. Second, the cutoff validation was conducted on a relatively small subset of samples, which may limit statistical power. Nonetheless, our findings are consistent with a recently published multicenter study^14^ demonstrating that the Lumipulse p‐tau217 assay reliably detects brain amyloid pathology. Additionally, our validation dataset was limited to amyloid status and p‐tau217 concentrations; demographic variables and relevant clinical confounders—such as kidney function^17^—were not available and therefore not accounted for in the analysis. Finally, we did not examine the susceptibility of the method to heterophilic antibody interference. Despite these limitations, the available data were sufficient to support cross‐validation of the two‐cutpoint classification model. We further conducted thorough validation experiments with the Lumipulse G assay and confirmed its robustness and suitability for routine implementation.

In conclusion, the Lumipulse G plasma p‐tau217 assay demonstrated good analytical performance and met pre‐specified key validation criteria, supporting its use in routine clinical practice—though caution is warranted when analyzing grossly hemolyzed samples. The application of pre‐established cutoffs was also verified, further supporting their integration into routine workflows. Nevertheless, the development of a CRM is urgently needed to enable assay standardization and ensure result traceability across platforms and laboratories, especially given the absolute concentration differences observed between assays.

## CONFLICT OF INTEREST STATEMENT

Kaj Blennow has served as a consultant and at advisory boards for Abbvie, AC Immune, ALZPath, AriBio, BioArctic, Biogen, Eisai, Lilly, Moleac Pte. Ltd, Neurimmune, Novartis, Ono Pharma, Prothena, Roche Diagnostics, Sanofi and Siemens Healthineers; has served at data monitoring committees for Julius Clinical and Novartis; has given lectures, produced educational materials and participated in educational programs for AC Immune, Biogen, Celdara Medical, Eisai and Roche Diagnostics; and is a co‐founder of Brain Biomarker Solutions in Gothenburg AB (BBS), which is a part of the GU Ventures Incubator Program, outside the work presented in this paper. Henrik Zetterberg has served at scientific advisory boards and/or as a consultant for Abbvie, Acumen, Alector, Alzinova, ALZPath, Annexon, Apellis, Artery Therapeutics, AZTherapies, CogRx, Denali, Eisai, Nervgen, Novo Nordisk, Optoceutics, Passage Bio, Pinteon Therapeutics, Prothena, Red Abbey Labs, reMYND, Roche, Samumed, Siemens Healthineers, Triplet Therapeutics, and Wave, has given lectures in symposia sponsored by Cellectricon, Fujirebio, Alzecure, Biogen, and Roche, and is a co‐founder of Brain Biomarker Solutions in Gothenburg AB (BBS), which is a part of the GU Ventures Incubator Program (outside submitted work). Nicholas J. Ashton has given lectures, produced educational materials, and participated in educational programs for Eli‐Lily, BioArtic, Alamar Biociences, Roche, and Quanterix and has served at scientific advisory boards and/or as a consultant for Abbvie, ALZpath, Beckman Coulter, New Amsterdam therapeutics, ImmunoBrain Theraptuics, Quanterix, Roche, Spear Bio. Silke Kern has served at scientific advisory boards, speaker and / or as consultant for Roche, Eli Lilly, Geras Solutions, Optoceutics, Biogen, Eisai, Merry Life, Triolab, Novo Nordisk, and Bioarctic, unrelated to present study content. Tevy Chan has served on the scientific advisory board for Eisai outside the scope of the present work. The other authors report no conflicts of interest. Any author disclosures are available in the supporting information.

## CONSENT STATEMENT

For the anonymized samples the collection at the Clinical Chemistry Laboratory, Sahlgrenska University Hospital, was conducted in accordance with the Ethics Committee at University of Gothenburg (EPN140811). The study was approved by the Regional Ethical Review Board in Gothenburg, Sweden, for the Gothenburg Birth Cohort studies H70 (Dnr 869‐13). Written informed consent was obtained from all participants and/or their legal representatives prior to inclusion in the study. All TRIAD participants, or their legal representatives, gave written informed consent, and ethical approval for the study was granted by the Montreal Neurological Institute PET Working Committee and the Douglas Mental Health University Institute Research Ethics Board (MP‐18‐2017‐157). All participants provided written informed consent, and the study was conducted in accordance with the Declaration of Helsinki.

## Supporting information



Supporting Information

Supporting Information
