# Supplementary material for: Analytical and clinical validation of the Lumipulse G plasma p‐tau217 assay for clinical implementation
Source: Alzheimers Dement. 2026 Apr 14;22(4):e71374. doi: 10.1002/alz.71374 (PMC13079079; doi:10.1002/alz.71374)
Supplement: Supplementary file 1 — Supporting Information [file ALZ-22-e71374-s001.docx]

Supplementary Material

**Analytical and clinical validation of the Lumipulse G plasma p-tau217 assay for clinical implementation**

Burak Arslan^1,2^, Johan Gobom^1,2^, Ulf Andreasson^1,2^, Laia Montoliu-Gaya^1^, Andrea L. Benedet^1^, Anna Dittrich^1,3^, Silke Kern^1,3^, Ingmar Skoog^1,3^, Nicholas J. Ashton^1,4,5^, Tevy Chan^6,7,8^, Nesrine Rahmouni^6,7^ , Pedro Rosa-Neto^6,9,10^, Kaj Blennow^1,2,11,12^, Henrik Zetterberg^1,2,13,14,15,16,17,18^, Hlin Kvartsberg^1,2^

**Affiliations:**

^1^ Department of Psychiatry and Neurochemistry, Institute of Neuroscience & Physiology, the Sahlgrenska Academy at the University of Gothenburg, Mölndal, Sweden

^2^ Clinical Neurochemistry Laboratory, Sahlgrenska University Hospital, Mölndal, Sweden

^3^ Department of Neuropsychiatry, Region Västra Götaland, Sahlgrenska University Hospital, Mölndal, Sweden

^4^ Banner Sun Health Research Institute, Sun City, AZ, USA.

^5^ Banner Alzheimer's Institute, Phoenix, AZ, USA.

^6^Translational Neuroimaging Laboratory, Montreal Neurological Institute-Hospital, Montreal, Canada

^7^ The Research Institute of the McGill University Health Centre, Montreal, Quebec, Canada

^8^ Division of Geriatric Medicine, Department of Medicine, McGill University, Montreal, Quebec, Canada

^9^Douglas Hospital Research Centre - Centre intégré universitaire de santé et services sociaux de l’Ouest-de-l’Île-de-Montréal, Verdun, Quebec, Canada

^10^ Peter O’Donnell Jr. Brain Institute (OBI), University of Texas Southwestern Medical Centre (UTSW), Dallas, USA

^11^ Paris Brain Institute, ICM, Pitié-Salpêtrière Hospital, Sorbonne University, Paris, France

^12^ Neurodegenerative Disorder Research Center, Division of Life Sciences and Medicine, and Department of Neurology, Institute on Aging and Brain Disorders, University of Science and Technology of China and First Affiliated Hospital of USTC, Hefei, P.R. China

^13^ Department of Pathology and Laboratory Medicine, University of Wisconsin School of Medicine and Public Health, Madison, WI, USA

^14^ Wisconsin Alzheimer’s Institute, School of Medicine and Public Health, University of Wisconsin, Madison, WI, USA

^15^ Department of Neurodegenerative Disease, Institute of Neurology, University College London, London, UK

^16^ UK Dementia Research Institute, University College London, London, UK

^17^ Hong Kong Center for Neurodegenerative Diseases, Hong Kong, China

^18^ Centre for Brain Research, Indian Institute of Science, Bangalore, India

#Correspondence to:

Burak Arslan, MD EuSpLM

Institute of neuroscience and physiology

Dept. Psychiatry and Neurochemistry

Sahlgrenska Academy at Gothenburg University

Mölndal Hospital, Hus V3, 43180 Mölndal, Sweden

E-mail: burak.arslan@gu.se

Tel: +46 723-661433

Supplementary Methods

S1. Interference - hemolysis

Two separate hemolysis interference experiments were conducted in this part of the study. In the first experiment, K_2_EDTA blood collection tubes were obtained from five individuals, with two tubes collected per individual. One tube was deliberately frozen prior to centrifugation to induce hemolysis, while the other was processed according to the standard protocol (centrifugation at 2000 × g for 10 minutes). The frozen tube was subsequently thawed at room temperature and centrifuged at 4000 × g for 10 minutes to yield hemolysate. Plasma from the non-hemolyzed tube was transferred to a secondary tube, aliquoted into three portions, and stored at −20 °C until analysis. One aliquot was used to determine the hemolysis, icterus, and lipemia (HIL) index, measured using the Roche Cobas platform. Another aliquot was spiked with 1% hemolyzed plasma to assess the effect of hemolysis under controlled lipemia conditions. Both hemolyzed and non-hemolyzed plasma samples from the same individuals were stored at −20 °C for at least 24 hours before analysis. On the day of analysis, all samples were thawed at room temperature. Prior to measurement, all aliquots were centrifuged at 2000 × g for 10 minutes. Spiked and unspiked aliquots were then analyzed for p-tau217 concentrations to evaluate the potential impact of hemolysis on assay performance.

In the second experiment, K_2_EDTA blood collection tubes (two per individual) were collected from 15 individuals using the same hemolysis induction and centrifugation protocols as in the first experiment. Based on findings from a prior experiment—where one sample with a high lipemia index showed a markedly elevated p-tau217 concentration after spiking with 1% hemolyzed plasma—only visually non-lipemic (clear, non-turbid) plasma samples with the lowest hemolysis and lipemia indices were selected for inclusion. Five such samples were chosen, and each was spiked with hemolyzed plasma at concentrations of 0.1%, 0.2%, 0.5%, 1%, and 2%.

In the second experiment, six patient plasma samples were analyzed both in their unspiked form and after being spiked with hemolyzed plasma obtained from a different individual. For each patient sample, plasma was aliquoted into six portions. One aliquot remained unspiked, while the remaining five were spiked with hemolyzed plasma at varying concentrations (0.1%, 0.2%, 0.5%, 1%, and 2%). All samples underwent the same centrifugation procedures as described in the first experiment. This experiment was designed to assess the dose-dependent impact of hemolysis on p-tau217 measurements. In the third experiment, a similar approach to the second experiment was employed, with three patient plasma samples spiked with hemolyzed plasma obtained from a different individual. This time, six spiking concentrations were tested: 0.05%, 0.1%, 0.2%, 0.5%, 1%, and 2%. A key difference in this experiment was the quantitative assessment of the HIL (hemolysis, icterus, lipemia) index for each spiked sample, which was measured using the Roche Cobas platform. Following this, p-tau217 concentrations were measured using the Lumipulse assay. This experiment aimed to further evaluate the sensitivity of the assay to varying levels of hemolysis and to correlate hemolysis index values with potential assay interference. In the fourth experiment, the same protocol as the first was used with blood collected from five individuals. The HIL index was determined for each sample, and 1% hemolyzed plasma was added to assess the effect under controlled lipemia conditions. In the fifth and final experiment, blood was collected from 15 individuals using the same hemolysis induction protocol as in the first experiment. Based on findings from the fourth experiment—where one sample with a high lipemia index showed a markedly elevated p-tau217 concentration after spiking with 1% hemolyzed plasma—only visually non-lipemic (clear, non-turbid) plasma samples with the lowest hemolysis and lipemia indices were included in the final experiment. Five such samples were selected, and each was spiked with hemolyzed plasma at concentrations of 0.1%, 0.2%, 0.5%, 1%, and 2%.

Supplementary Results

Supplementary Table 1. Assay and analytical characteristics of the Lumipulse G p-tau217

|  | **p-tau 217 Lumipulse** |
| --- | --- |
| Commercial/in-house | Commercial (Fujirebio, Japan), RUO |
| Product number | 81472 |
| Lot number | Cartridge Lot Number: 5023 |
|  | Substrate Lot Number : 5027 |
| Instrument | LUMIPULSE® G1200 |
| Detection system/technology | Luminescence (ALP + AMPPD) |
|  | CLEIA |
|  | Two-step |
| Sample volume(μL) - 1 replicate | 200 |
| Sample volume(μL) - 2 replicate | 300 |
| Sample dilution | No dilution |
| Measurement range (pg/mL) | 0.030-10 |
| Intra-assay CV%, Low QC, High QC | 3.9 , 3.6 |
| Inter-assay CV%, Low QC, High QC | 3.9 , 3.6 |
| Clinical samples CV% (duplicate measurement) | 6.42 |
| Capture antibody | RD-85 |
|  | (epitope phosphorylated at T217) |
| Detector antibody | BT2 (epitope 194-198) and |
|  | HT7 (epitope 159-163) |
| Calibrator | pTau 217 synthetic peptid |
|  |  |
| Analytical LLoQ (pg/mL) | 0.03* |

Overview of assay characteristics for the Lumipulse G plasma p-tau217 assay. Key specifications are summarized, including platform type, detection technology, antibodies, calibrators, sample input requirements, and precision metrics. Antibody and calibrator details were obtained from previously published sources.

*The reported analytical LLoQ for the Lumipulse assay reflects the manufacturer’s claimed value.

*Abbreviations: RUO – Research use only, ALP – Alkaline phosphatase, AMPD – 2-Amino-2-methyl-1-propanol , CLEIA – Chemiluminescent enzyme immunoassay , CV – Coefficient of variation, QC – Quality control, LLoQ – Lower limit of quantification*

Supplementary Table 2. Preparation of spiking materials for specificity experiment

| Spiking material | Dilution | C (pg/uL) |
| --- | --- | --- |
| 1 | Only diluent |  |
| 2 | 1:20 | 0.05 |
| 3 | 1:10 | 0.1 |
| 4 | 1:4 | 0.25 |
| 5 | 1:2 | 0.5 |
| 6 | 1 | 1 |

A stock solution of non-phosphorylated full-length recombinant Tau-441 protein was prepared at a concentration of 1 pg/µL. Serial dilutions were subsequently performed using the Lumipulse sample diluent to obtain a range of spiking concentrations, as presented in the table. For each condition, 5 µL of the diluted spiking material was added to 250 µL of plasma, and the resulting mixtures were used in the subsequent experiment described in Table 2.

Supplementary Table 3. Overview of the hemolysis experiments

| **Experiment 1** | **p-tau217 (pg/mL)** | **Relative difference -%** | **H** | **I** | **L** |
| --- | --- | --- | --- | --- | --- |
| Sample 1 | 0.173 |  | 66 | 18 | 1 |
| Sample 1 - 1% H | 1.303 | 653% | 84 | 125 | 1 |
| Sample 2 | **0.061** |  | 12 | 7 | 1 |
| Sample 2 - 1% H | 0,067 | 10% | 20 | 108 | 1 |
| Sample 3 | **0.083** |  | 11 | 10 | 1 |
| Sample 3 - 1% H | 0.07 | -16% | 16 | 124 | 1 |
| Sample 4 | **0.068** |  | 11 | 3 | 1 |
| Sample 4 - 1% H | 0.082 | 21% | 13 | 102 | 1 |
| Sample 5 | 0.188 |  | 29 | 8 | 0 |
| Sample 5 - 1% H | 0.185 | -2% | 33 | 116 | 0 |
| **Experiment 2** | **p-tau217 (pg/mL)** |  | **H** | **I** | **L** |
| Sample 1 | 0.126 |  | 6 | 0 | 19 |
| Sample 1 - 0.125% H | 0.124 | -1.6% |  |  |  |
| Sample 1 - 0.25% H | 0.121 | -4.0% |  |  |  |
| Sample 1 - 0.5% H | 0.135 | 0.009 |  |  |  |
| Sample 1 - 1% H | 0.133 | 0.007 |  |  |  |
| Sample 1 - 2% H | 0.129 | 0.003 |  |  |  |
| Sample 2 | **0.119** |  |  |  |  |
| Sample 2 - 0.125% H | 0.126 | 0.007 | 11 | 1 | 10 |
| Sample 2 - 0.25% H | 0.122 | 0.003 |  |  |  |
| Sample 2 - 0.5% H | 0.128 | 0.009 |  |  |  |
| Sample 2 - 1% H | 0.123 | 0.004 |  |  |  |
| Sample 2 - 2% H | 0.126 | 0.007 |  |  |  |
| Sample 3 | **0.116** |  | 17 | 0 | 19 |
| Sample 3 - 0.125% H | 0.114 | -0.002% |  |  |  |
| Sample 3 - 0.25% H | 0.111 | -0.005% |  |  |  |
| Sample 3 - 0.5% H | 0.105 | -0.011 |  |  |  |
| Sample 3 - 1% H | 0.11 | -0.006 |  |  |  |
| Sample 3 - 2% H | 0.125 | 0.009 |  |  |  |
| Sample 4 | 0.242 |  | 15 | 1 | 11 |
| Sample 4 - 0.125% H | 0.243 | 0.001 |  |  |  |
| Sample 4 - 0.25% H | 0.246 | 0.004 |  |  |  |
| Sample 4 - 0.5% H | 0.245 | 0.003 |  |  |  |
| Sample 4 - 1% H | 0.256 | 0.014 |  |  |  |
| Sample 4 - 2% H | 0.251 | 0.009 |  |  |  |
| Sample 5 | 0.06 |  | 12 | 1 | 7 |
| Sample 5 - 0.125% H | 0.069 | 0.009 |  |  |  |
| Sample 5 - 0.25% H | 0.072 | 0.012 |  |  |  |
| Sample 5 - 0.5% H | 0.075 | 0.015 |  |  |  |
| Sample 5 - 1% H | 0.079 | 0.019 |  |  |  |
| Sample 5 - 2% H | 0.084 | 0.024 |  |  |  |

This table presents the HIL index along with results from two independent hemolysis experiments, each using different sets of samples. In the first experiment, five samples were tested, and only 1% hemolysate was spiked into the native plasma. For each sample, relative differences were calculated and reported as percentages. In the second experiment, five additional samples were tested, each spiked with a range of hemolysate concentrations (0.125%, 0.25%, 0.5%, 1.5%, and 2%) in their corresponding native plasma. Relative differences were again presented as percentages. Unspiked samples with concentrations below the LLoQ (0.12 pg/mL) are shown in bold.

Supplementary table 4. Demographics and clinical characteristics of the TRIAD cohort

| **Characteristic** | **CU-**  N = 10^1^ | **CU+**  N = 10^1^ | **MCI+**  N = 27^1^ | **AD**  N = 4^1^ | **MCI-**  N = 3^1^ | **p-value**^2^ |
| --- | --- | --- | --- | --- | --- | --- |
| Sex |  |  |  |  |  | 0.7 |
| Female | 5 (50%) | 5 (50%) | 11 (41%) | 3 (75%) | 2 (67%) |  |
| Male | 5 (50%) | 5 (50%) | 16 (59%) | 1 (25%) | 1 (33%) |  |
| Age | 69.3 (68.1–71.0) | 68.9 (66.5–76.6) | 74.6 (71.3–77.6) | 70.0 (65.8–76.9) | 73.9 (69.2–75.3) | 0.080 |
| YOE | 18.0 (15.0–20.0) | 14.5 (13.0–17.0) | 17.0 (15.0–18.0) | 13.5 (13.0–14.5) | 13.0 (11.0–15.0) | 0.057 |
| MMSE | 30.0 (29.0–30.0) | 30.0 (29.0–30.0) | 28.0 (27.0–29.0) | 23.0 (19.0–28.0) | 30.0 (29.0–30.0) | <0.001 |
| Missing | 0 | 1 | 0 | 1 | 0 |  |
| APOE - ε4 |  |  |  |  |  | 0.082 |
| Non-carriers | 9 (90%) | 5 (50%) | 11 (41%) | 2 (50%) | 2 (67%) |  |
| Carriers | 1 (10%) | 5 (50%) | 16 (59%) | 2 (50%) | 1 (33%) |  |
| Amyloid PET, SUVR | 1.26 (1.23–1.28) | 1.88 (1.80–2.09) | 2.50 (2.09–2.74) | 2.55 (2.12–2.73) | 1.18 (1.18–1.34) | <0.001 |
| Tau PET, SUVR (meta-ROI) | 0.94 (0.88–1.03) | 0.98 (0.86–1.22) | 1.42 (1.08–1.78) | 2.37 (1.72–3.27) | 0.96 (0.82–1.06) | <0.001 |
| ^1^n (%); Median (Q1–Q3) | | | | | | |
| ^2^Fisher's exact test; Kruskal-Wallis rank sum test; NA  *Abbreviation : CU−, cognitively unimpaired amyloid-negative; CU+, cognitively unimpaired amyloid-positive; MCI+, mild cognitive impairment amyloid-positive; MCI−, mild cognitive impairment amyloid-negative; AD, Alzheimer’s disease dementia; YOE, years of education; MMSE, Mini-Mental State Examination; APOE ε4, apolipoprotein E epsilon 4 allele; PET, positron emission tomography; SUVR, standardized uptake value ratio* | | | | | | |

Supplementary Figure 1. Run-to-Run reproducibility of p-tau217 measurements using Lumipulse assay


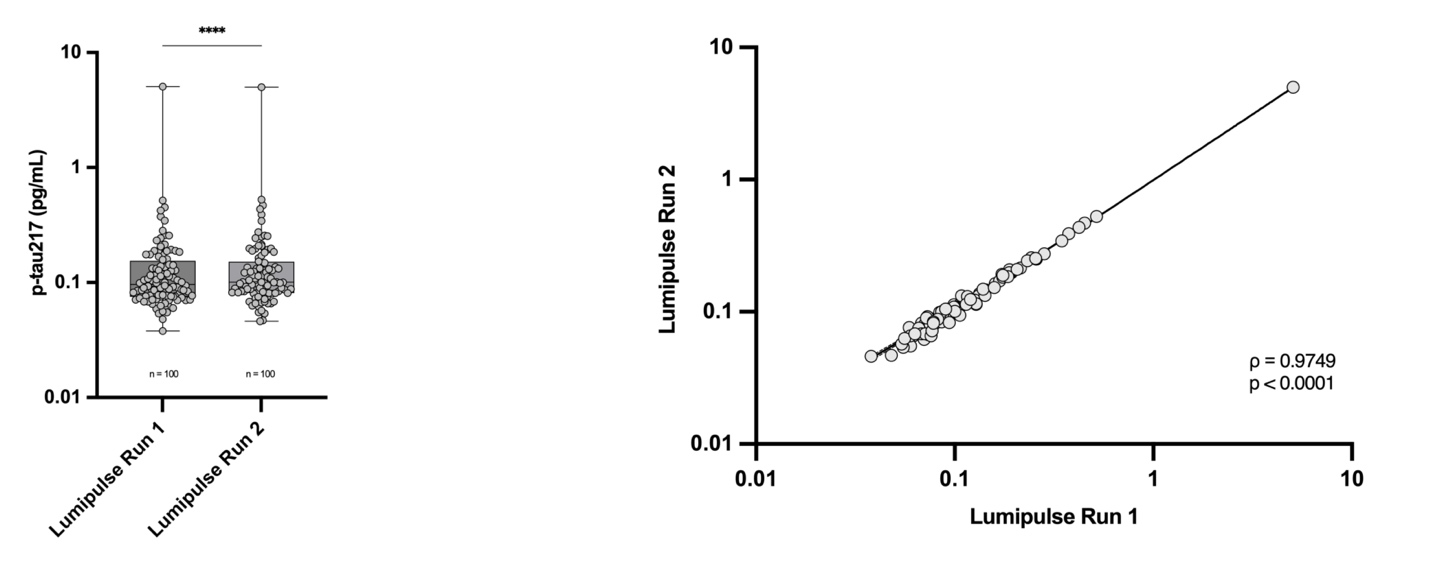


Comparison of p-tau217 concentrations measured in two independent runs using the Lumipulse assay. For this assay, 100 plasma samples were analyzed in two separate runs to assess run-to-run variability. The time interval between the runs was one week for the Lumipulse assay. High correlation was observed between the runs (Lumipulse: ρ = 0.9749, p < 0.0001). Data are presented on a log scale for Lumipulse. Paired comparisons between runs were assessed, and significance is indicated (**** p < 0.0001).

Supplementary Figure 2. Intra-Assay precision of Lumipulse p-tau217 immunoassays

across a range of concentration


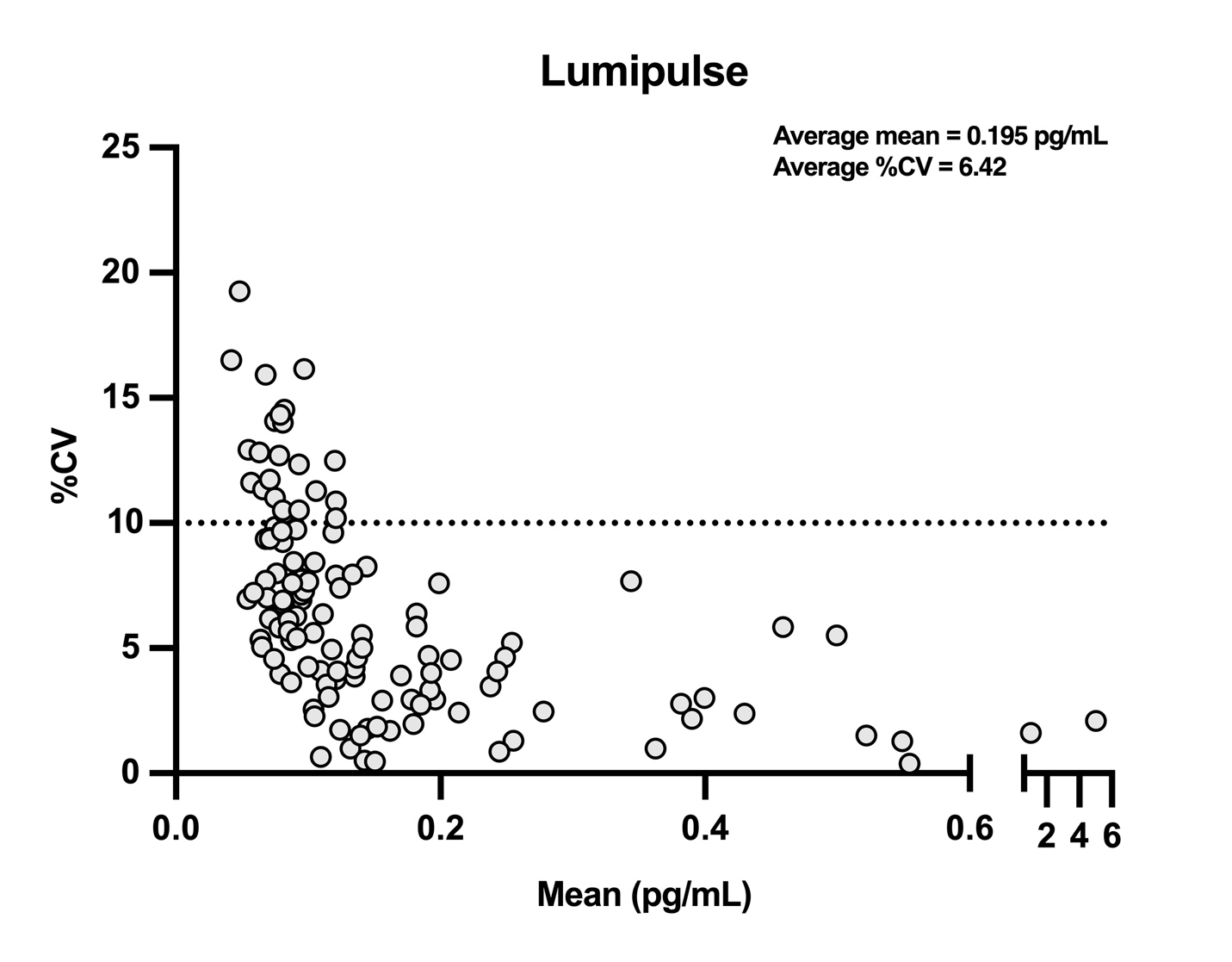


Intra-assay coefficient of variation (%CV) plotted against the mean p-tau217 concentration for Lumipulse G platform. Each point represents a distinct sample measured in duplicate within the same assay run. The average %CV was 6.42% for Lumipulse (average mean: 0.195 pg/mL), indicating acceptable precision across assays.

*Abbreviations: %CV = percent coefficient of variation; p-tau217 = phosphorylated tau at threonine 217*

Supplementary Figure 3. Comparative analysis of p-tau217 concentrations measured across two separate runs (reference – validation) in cutoffs validation part of the study


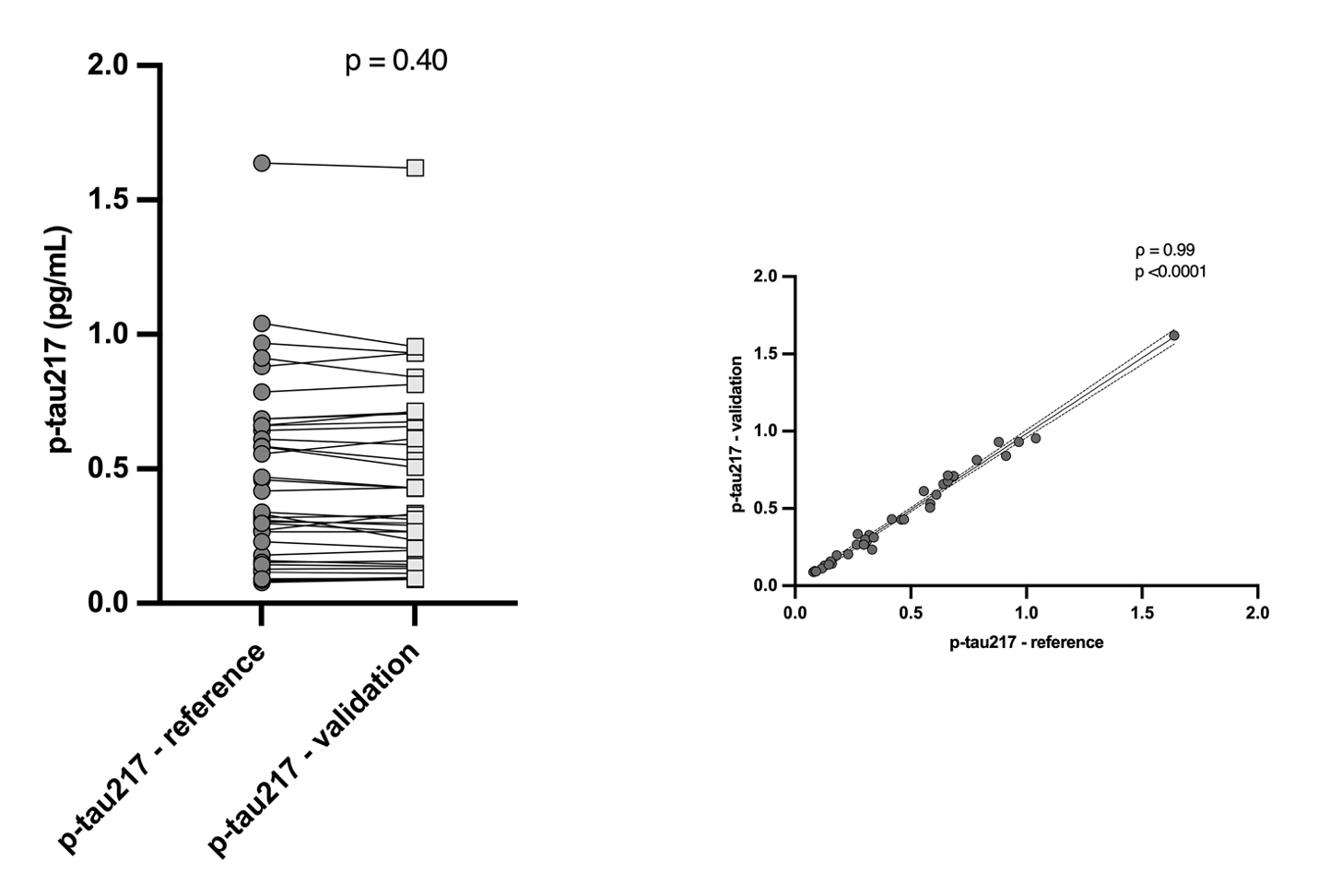


On the left - Box plot comparison shows no statistically significant difference in p-tau217 concentrations between the reference and validation runs (Wilcoxon matched-pairs test, p = 0.40). Median values were nearly identical (0.34 pg/mL vs. 0.33 pg/mL), and the variability remained consistent across runs.

On the right - Spearman correlation analysis demonstrated excellent agreement between runs (r = 0.99, p < 0.0001), supporting the high reproducibility of the assay under routine analytical conditions
